# Medical Items

**Published:** 1912-01

**Authors:** 


### MEDICAL ITEMS.

### THE APPETITE IN TUBERCULOSIS.

In view of the fact that hypernutrition, or so-called forced feeding constitutes one of the important indications in the treatment of many cases of tuberculosis, more than ordinary attention must always be devoted to maintaining the appetite. Unfortunately, many of these patients have an aversion to the very foods which are best adapted for repairing and resisting the ravages of the disease. It is here that Gray's Glycerine Tonic Comp. serves one of its most important purposes, by reason of its notable capacity to awaken a deficient appetite in a perfectly natural manner. It not only possesses the desirable feature of great palatability, but through its tonic properties, it never fails to impart just the right tone to the digestive organs. Thus the effects are so much more permanent and far reaching than are obtained from ordinary stomachics, that not only are larger quantities of nourishment freely taken by the patient, but a crorespondingly increased amount finds its way to the remote tissues.

